# Has the practice of radiation oncology for locally advanced and metastatic non-small-cell lung cancer changed in Canada?

**DOI:** 10.3747/co.v17i1.387

**Published:** 2010-02

**Authors:** K. Han, A. Bezjak, W. Xu, G. Kane

**Affiliations:** * Department of Radiation Oncology, Princess Margaret Hospital, University Health Network, Toronto, ON; † Department of Biostatistics, University Health Network, Toronto, ON; ‡ Department of Radiation Oncology, University of Washington Medical Center, Seattle, WA, U.S.A

**Keywords:** Non-small-cell lung cancer, palliation, radiation, survey

## Abstract

**Aim:**

Previous surveys have revealed wide variations in the management by radiation oncologists of non-small-cell lung cancer (nsclc) in Canada. The aim of the present study was to determine the current patterns of practice for locally advanced and metastatic nsclc among Canadian radiation oncologists.

**Materials and Methods:**

An online survey was distributed electronically to all members of the Canadian Association of Radiation Oncologists. Those who treat lung cancer were invited to participate. The survey consisted of three scenarios focusing on areas of nsclc treatment in which the radiotherapy (rt) regimen that provides the best therapeutic ratio is unclear.

**Results:**

Replies from 41 respondents were analyzed. For an asymptomatic patient with stage iiib nsclc unsuitable for radical treatment, 22% recommended immediate rt, and 78% recommended rt only if the patient were to become symptomatic. Those who believed that immediate rt prolongs survival were more likely to recommend it (*p* = 0.028). For a patient with a bulky stage iiib tumour and good performance status, 39% recommended palliative treatment, and 61% recommended radical treatment (84% concurrent vs. 16% sequential chemoradiation at 60–66 Gy in 30–33 fractions). Those who believed that chemoradiation has a greater impact on survival were more likely to recommend it (*p* < 0.001). For a symptomatic patient with stage iv nsclc, 54% recommended external-beam rt (ebrt) alone, 41% recommended other modalities (brachytherapy, endobronchial therapy, or chemotherapy) with or without ebrt, and 5% recommended best supportive care. A majority (76%) prescribed 20 Gy in 5 fractions for ebrt.

**Conclusions:**

Compared with previous surveys, more radiation oncologists now offer radical treatment for locally advanced nsclc. Management of nsclc in Canada may be evidence-based, but perception by radiation oncologists of the treatment’s impact on survival also influences treatment decisions.

## 1. INTRODUCTION

Lung cancer is one of the most common causes of cancer mortality in both men and women worldwide 1. Most patients develop non-small-cell lung cancer (nsclc) and have either locally advanced or metastatic disease at presentation. Previous surveys conducted in 1993 and 1995 revealed wide variations in the treatment policies of Canadian radiation oncologists for nsclc, and those surveys concluded that personal beliefs, rather than universal knowledge, tended to guide the management of nsclc in Canada 2,3.

In the 13 years since the more recent of the two surveys, the results of several randomized clinical trials focusing on palliative radiotherapy (rt) for locally advanced and metastatic nsclc have been published 4–12. However, the optimal dose, fractionation scheme, and timing for palliative rt in nsclc remain controversial. The present study was undertaken to determine current patterns of practice across Canada for locally advanced and metastatic nsclc and to establish whether practice actually changed in the preceding decade.

The study survey consisted of three case scenarios designed to question radiation oncologists on areas of nsclc treatment in which the rt regimen that provides the best therapeutic ratio is unclear (Table I).

## 2. MATERIALS AND METHODS

An online survey was distributed electronically to all active members of the Canadian Association of Radiation Oncologists (*n* = 274) in March 2007; those treating lung cancer were invited to participate. The study was approved by the Research Ethics Board at the University Health Network (Toronto, ON).

The initial page of the online survey required the explicit consent of study participants before the survey started. Respondents were presented with the three case scenarios listed in Table I. For each scenario, they were asked to specify the treatment regimen they would use, details pertaining to treatment delivery, and the effect that the treatment might potentially have on patient survival. In addition, non-identifying demographic information was solicited: the location and year in which respondents completed their specialty training, their current location and type of practice, and their level of experience in treating lung cancer. Finally, respondents were asked to rank the effect of each of the following factors on their treatment decisions: journals and books, seminars and meetings, practice guidelines, departmental policy, colleague preferences and expert opinions, personal experience, and availability of open clinical trials.

Responses were collected as either the single best answer or all applicable answers (chosen from menu options) and as free text. Percentages of respondents selecting a specific option were calculated. Statistical comparisons were made using contingency tables, generally with a chi-square test, using a Yates correction when required. When answer groups included fewer than 20 respondents or fewer than 5 events, the Fisher exact test was employed. Logistic regression was used to identify demographic factors associated with the treatment choice in each case. All tests of significance were two-sided, and differences were considered statistically significant at *p* values below 0.05. The SPSS software application (version 14: SPSS, Chicago, IL, U.S.A.) was used for all analyses.

## 3. RESULTS

### 3.1 Overview

Of the 46 responses collected, 41 were sufficiently complete and suitable for analysis; 5 were incomplete (>95% of questions unanswered) and were therefore excluded. This sample represented responses from 23 of 37 Canadian cancer centres and from approximately 30%–40% of the radiation oncologists treating lung cancer in Canada, based on a previous survey that identified 103 radiation oncologists treating lung cancer patients in Canada 13 and estimating that that number had grown by 10%–20% in the intervening period. All regions of the country were well represented. The distribution of respondents by centres showed 1 respondent from each of 15 centres, 2 respondents from 4 centres, 3 respondents from 1 centre, 4 respondents from 1 centre, and 5 respondents from 2 centres. Table II summarizes demographic data for the respondents.

### 3.2 Scenario A: Asymptomatic Patient with Stage IIIB NSCLC Unsuitable for Radical Treatment

#### 3.2.1 Radiotherapy Plan

For an asymptomatic patient with stage iiib nsclc unsuitable for radical treatment, 22% of respondents recommended immediate rt, and 78% recommended rt only if the patient became symptomatic (Table III). Of respondents choosing immediate rt, all but 1 (89%) aimed to prevent symptoms (Table IV). In all instances, computed tomography (ct) simulation was chosen. All respondents but 1 (89%) chose an anterior–posterior parallel pair (ap/pa) plan over a three-dimensional (3D) conformal plan. Three dose fractionation schedules were prescribed: 20 Gy in 5 fractions over 1 week (4 respondents); 30 Gy in 10 fractions over 2 weeks (3 respondents); and 60 Gy in 30 fractions over 6 weeks (1 respondent). One respondent did not specify a dose–fractionation scheme. Half the respondents predicted that the average wait time for the start of rt treatment (once the decision to treat was made) would be less than 1 week. Respondents in departments with wait times exceeding 1 week were more likely to chose a shorter fractionation schedule (*p* = 0.029).

#### 3.2.2 Prognosis and Effect of Treatment

A majority of respondents (61%) estimated the median survival of the patient in scenario A to be 6–12 months (Table IV). Only 27% of the respondents believed that immediate rt (versus delayed rt until needed to treat symptoms) would prolong survival. Those who believed that immediate rt would prolong survival were more likely to recommend it (*p* = 0.028).

### 3.3 Scenario B: Patient with Bulky, Unresectable Stage IIIB NSCLC and Good Performance Status

#### 3.3.1 Radiotherapy Plan

For a patient with a bulky, stage iiib tumour and good performance status (ps), 39% of respondents recommended palliative treatment, and 61% recommended radical treatment (Table IV). Most respondents (76%) recommended combined chemoradiation, and 20% recommended rt alone (Table III). Those who chose radical chemoradiation preferred a concurrent over a sequential approach (84% vs. 16%). A dose range of 60–66 Gy in 30–33 fractions was prescribed by all respondents but 1 (who recommended radical treatment, Table V). For palliative treatment, the most frequently prescribed dose was 30 Gy in 10 fractions over 2 weeks. Overall, 54% of the respondents aimed to prolong survival; 37%, to relieve symptoms; 7%, to prevent symptoms; and 2%, to “provide local control” (Table IV). Respondents who aimed primarily to relieve symptoms selected a lower dose and a shorter fractionation schedule (*p* < 0.001). Simulation by ct was chosen over fluoroscopic simulation by all respondents but 1. The most commonly chosen plan was a 3D conformal plan (56%), with 31% of the respondents electing to use an ap/pa plan, and the remaining 13% choosing an oblique or other plan (unspecified). A majority of respondents (59%) predicted that the average wait time for starting rt treatment would be 1–2 weeks.

#### 3.3.2 Prognosis and Effect of Treatment

Of the survey respondents, 59% estimated the median survival of the patient in scenario B to be 6–12 months (Table IV). A preponderance believed that treatment would prolong survival (88% stated that rt would prolong survival, 64% that chemotherapy would, and 93% that chemoradiation would). Respondents who recommended radical treatment or chemoradiation (as compared with those who did not) indicated a greater effect for chemoradiation on survival (*p* < 0.001).

### 3.4 Scenario C: Symptomatic Stage IV Patient with Poor Performance Status

#### 3.4.1 Radiotherapy Plan

For a symptomatic patient with stage iv nsclc, 54% recommended external-beam rt (ebrt) alone, 10% recommended endobronchial therapy followed by ebrt, 5% recommended brachytherapy followed by ebrt, 2% recommended brachytherapy alone, 24% recommended ebrt followed by chemotherapy, and 5% recommended best supportive care (Table III). All respondents aimed to relieve symptoms. Most (82%) chose ct simulation over fluoroscopic simulation (18%), and an ap/pa plan (95%) over a 3D conformal plan (5%). Five dose–fractionation schedules for ebrt were prescribed (*n* = 38): 20 Gy in 5 fractions over 1 week (76%); 30 Gy in 10 fractions over 2 weeks (13%); 36 Gy in 12 fractions over 2.5 weeks (8%); and 17 Gy in 2 fractions over 1 week (3%). Each respondent who chose brachytherapy prescribed a different dose: 8 Gy every week for 3 weeks, and single 10-Gy and single 20-Gy fractions. Most respondents (79%) predicted that the average wait time for rt treatment start would be less than 1 week (Table IV). Respondents in departments with wait times exceeding 1 week tended to chose a shorter fractionation schedule (*p* = 0.016).

#### 3.4.2 Prognosis and Effect of Treatment

Most respondents (71%) estimated the median survival of the patient in scenario C to be less than 6 months, and none felt that it would be longer than 18 months (Table IV). Almost one third of the respondents (32%) believed that rt would prolong survival; 66%, that chemotherapy would prolong survival; and 51%, that chemoradiation would prolong survival. Respondents who recommended chemotherapy (as compared with those who did not) indicated a greater effect for chemotherapy on survival (*p* < 0.001).

### 3.5 Correlation with Demographic Factors

We also examined demographic factors of the respondents possibly associated with treatment choice in each case: the year specialty training was completed, the location of residency or fellowship training, number of years dealing with lung cancer, location of practice, type of practice, and number of new nsclc patients seen annually.

In scenario A, radiation oncologists in the community were more likely than those in university-affiliated centres to recommend immediate rt (*p* = 0.011). In scenario B, radiation oncologists who had finished training more recently and those who saw more patients per year were more likely to recommend radical treatment (*p* = 0.017 and *p* = 0.010 respectively). We observed no correlations between treatment choice and demographic factors in scenario C.

When asked to rank the effect of 7 factors with respect to treatment decisions, 17 respondents chose practice guidelines as the most important, 9 chose availability of open clinical trials, 4 chose departmental policy, 4 chose journals and books, 3 chose seminars and meetings, 3 chose personal experience, and 1 chose colleague preferences and expert opinions. “Practice guidelines” were ranked by 80% of the respondents as one of the three most important factors influencing their treatment decisions; “personal experience” was ranked as one of the three most important factors by 52%.

## 4. DISCUSSION

Palliative rt regimens in advanced nsclc have been subject to rigorous evaluation in clinical trials 4–12,14. However, the way in which radiation oncologists interpret the results of these trials influences their treatment recommendations. The present study explored current patterns of practice for locally advanced and metastatic nsclc in Canada. Our survey had an estimated response rate of approximately 30%–40%. Although limited, our response rate is higher than that in other similar studies 15,16. A precise response rate cannot be provided, because it is difficult to determine the exact number of radiation oncologists who were treating lung cancer in Canada at the time of the survey. A better way to investigate patterns of practice across a nation is through practice audit; however, that approach is time consuming and often impractical. As a result, many studies, including ours, use survey data as a surrogate. One potential limitation is that survey data may not accurately represent what is done in actual practice.

With advancements in technology, changes have occurred in how palliative rt is delivered. Our survey confirmed these changes. In all three cases, ct simulation was used by most respondents (82%– 100%). However, for “palliative” cases (scenarios A and C), ap/pa was preferred over a conformal plan (89%–95%). That finding is consistent with an ap/pa plan being unlikely to exceed spinal cord tolerance for palliative cases, but not for radical cases.

For minimally symptomatic patients with unresectable nsclc not suitable for radical treatment (such as in scenario A), a randomized trial published in 2002 showed that immediate rt does not improve symptom control, quality of life, or survival when compared with delay until symptoms require treatment 17. The median survival of patients in this trial by Falk *et al.* were 8.3 months (immediate rt) and 7.9 months (delayed rt—most did not receive rt). In our study, most respondents estimated the survival of the patient in scenario A to be 6–12 months, which agrees with the published results. Most respondents also believed that immediate rt would not prolong survival, and 78% chose not to give rt immediately and to treat only if the patient became symptomatic. When a similar case was presented in a survey conducted in 1995, only 17% of the respondents chose no immediate active treatment 3. This change in the reported practice pattern may indeed be attributable to the published evidence. Increased use of chemotherapy may be a factor as well.

Scenario B is the most challenging scenario with respect to knowing the best evidence-based treatment. It was purposely designed to address a specific dilemma: What is the chance of cure for a patient with good ps, a bulky tumour, and no obvious metastases, if treated with radical chemoradiation rather than high-dose palliative rt?

Several meta-analyses have shown that chemoradiation improves survival when compared with rt alone for unresectable stage iii nsclc (absolute survival benefit of 2 months or 2% at 5 years) 18–20. However, the entry criteria for each trial differed, and the trials often do not explicitly state the tumour size, bulk, or intrathoracic disease deemed suitable for radical treatment. Most studies excluded patients based only on their blood work, pulmonary function, weight loss, and performance status (Eastern Cooperative Oncology Group score >1 or >2). The patient in scenario B certainly could have been an eligible candidate for randomized trials that investigated radical chemoradiation for unresectable stage iii nsclc. In most of those trials, the median survival of patients with unresectable stage iii nsclc who received chemoradiation ranged from 10–18 months, compared with 9–12 months with radiotherapy alone 18–20, and approximately 8 months with no immediate treatment (reported by Falk *et al.*) 17. The estimate of the respondents concerning the effect of chemoradiation on survival for the patient in scenario B varied. However, as in scenario A, the estimate given by most respondents for the median survival of patient B without treatment agrees with the results in the existing literature and is also consistent with their estimates in scenario A. When a case similar to that of the patient in scenario B was presented in a previous survey in 1993 2, but with a much smaller tumour (4 cm compared with the 9 cm here) and a younger patient (59 years vs. 72 years), only 31% of the respondents chose a radical approach. In contrast, 61% of the respondents in the present study chose a radical approach despite a much larger tumour (and thus likely a lower chance of cure), and more advanced patient age. Compared with past practice, the practice of today’s Canadian radiation oncologists has evolved to favour more aggressive treatment for nsclc. However, whether this evolving practice is evidence-based is less clear.

Despite numerous studies examining various palliative dose–fractionation regimens, the optimal regimen for a symptomatic patient such the one described in scenario C is not clearly established. The most recent Cochrane review concluded that most patients with locally advanced nsclc and thoracic symptoms should be treated with short courses of palliative rt, but selected patients with good ps should be considered for higher-dose palliative regimens— for example, 36 Gy in 12 fractions) 14. Practice patterns tend to be geographically based, and studies conducted by national groups often influence practice more than do studies undertaken elsewhere. The study most relevant to Canadians is the National Cancer Institute of Canada Clinical Trials Group sc.15 trial, in which 20 Gy in 5 fractions provided not only a similar degree of palliation of thoracic symptoms but also a 2-month survival advantage as compared with a single fraction of 10 Gy 8. On post-hoc subgroup analysis, the improvement persisted only for patients who were ps 0–1 and had localized disease. Overall, patients who received 20 Gy in 5 fractions experienced significantly greater improvement in symptoms related to lung cancer, pain, and ability to carry out normal activities and a better global quality of life.

In the present survey, for the patient in scenario C with a ps 2, most respondents (76%) prescribed 20 Gy in 5 fractions using ebrt, and 71% of those who prescribed 20 Gy in 5 fractions believed that this regimen had no effect on survival, in keeping with results from the Canadian study. A majority of respondents (66%) also believed that chemotherapy had some effect on survival, and 24% chose ebrt followed by chemotherapy. Those numbers are similar to the numbers obtained in the previous survey (60% and 20% respectively). However, it is hard to make a direct comparison, because the patient in the present scenario is considerably older and has a poorer ps and more medical co-morbidities. For patients with a good ps, the meta-analysis from 1996 showed that, as compared with best supportive care, chemotherapy is associated with improved quality of life and survival (5–7.5 months vs. 4 months) 19. This meta-analysis is being updated, and the benefit of chemotherapy is expected to be even greater now with third-generation regimens.

Data are also available to support the use of single-agent chemotherapy in elderly patients, or patients with a ps of 2 21,22, but the evidence is not abundant for elderly patients with a ps of 2 because they are often underrepresented in clinical trials 23. In the present study, most respondents (71%) estimated the median survival of the patient in scenario C to be less than 6 months, and they believed that chemotherapy would provide a survival benefit of less than 3 months, in keeping with the available evidence. Only 1 respondent chose brachytherapy alone, because there is evidence that high dose rate (hdr) brachytherapy alone is less effective than ebrt alone 24,25. Existing evidence does not conclusively indicate that hdr brachytherapy and ebrt would provide improved symptom relief over ebrt alone, and only 2 respondents (5%) chose brachytherapy with ebrt 24.

Endobronchial therapy followed by ebrt was chosen by 4 respondents (10%). Although the role of endobronchial therapy such as photodynamic therapy is not well defined, it can contribute to symptom relief. One randomized trial showed significant improvement in the control of hemoptysis and the relief of dyspnea for patients receiving photodynamic therapy plus rt as compared with those receiving rt alone 26. For scenario C, most of the recommendations are in keeping with available evidence.

Overall, most Canadian radiation oncologists appear to practice evidence-based medicine, because their regimens are in agreement with results of several randomized studies. However, as in the preceding decade 3, the beliefs of the radiation oncologists about the treatment’s effect on survival still vary and also influence treatment decisions. For each scenario in the present study, radiation oncologists were more likely to recommend the treatment that they believed would have the greater impact on survival. Survival benefits may be overestimated by some of the respondents. The effect of the beliefs of the oncologists was also illustrated by Graham *et al.*, who found that the attitudes of Ontario oncologists toward practice guidelines correlated with their intention to use those guidelines 27. Therefore, if strategies aiming to increase the uptake of evidence into practice are to be useful, they need to address those beliefs and perceptions.

## 6. CONFLICT OF INTEREST

There are no potential conflicts of interest.

## Figures and Tables

**TABLE I:** Three case scenarios of non-small-cell lung cancer (nsclc) presented in the survey

*Scenario A*	*Should patients with locally advanced*nsclc*unsuitable for resection or radical radiotherapy, and with no or minimal thoracic symptoms, be given palliative radiotherapy (*rt*) immediately or as needed to treat symptoms?* An 80-year-old man with chronic obstructive pulmonary disease and chronic renal failure is found to have a 4-cm mass in the right upper lobe on routine chest radiograph. Biopsy of the mass confirms the diagnosis of squamous cell carcinoma (scc). Mediastinoscopy reveals bilateral mediastinal lymph node involvement with scc. The rest of the metastatic workup is negative. Aside from fatigue, the patient has only a minor chronic cough, an Eastern Cooperative Oncology Group score of 2, and 14% weight loss within the past 6 months.
*Scenario B*	*Should patients with unresectable stage*iii nsclc*and large tumour volume be considered for radical treatment?* A 72-year-old previously healthy woman presents with a 2-week history of minor hemoptysis and no other symptoms. Her chest radiograph shows a 9-cm mass in the right upper lobe and associated partial atelectasis. Biopsy of the mass confirmsthe diagnosis of squamous cell carcinoma (scc). Mediastinoscopy reveals bilateral bulky mediastinal lymph node involvement with scc, and the rest of the metastatic workup is negative. Her forced expiratory volume in 1 s is 2 L (80% of predicted). She has mild fatigue, an Eastern Cooperative Oncology Group score of 1–2, and 3% weight loss within the past 3 months.
*Scenario C*	*For symptomatic patients with metastatic*nsclc*, which dose–fractionation regimen is most appropriate?* A 70-year-old man presents with worsening dyspnea and no other symptoms. Computed tomography imaging of the chest shows a mass measuring approximately 4 cm and associated right middle lobe (rml) atelectasis. Bronchoscopy reveals the mass to be almost completely obstructing the diagnosis the rml bronchus, and biopsy of the mass confirms of squamous cell carcinoma. Bone scan shows 2 foci of metastatic deposits in the spine, which are asymptomatic. The patient has multiple co-morbidities, including diabetes and coronary artery disease, and has an Eastern Cooperative Oncology Group score of 2–3 and 12% weight loss within the past 6 months.

**TABLE II:** Characteristics of the survey respondents

Characteristic	Respondents	
	(*n*)	(%)
All respondents	41	100
Year specialty training completed		
1970–1979	1	2
1980–1989	8	20
1990–1999	18	44
2000 and after	14	34
Location of specialty training		
Canada	38	93
Britain	3	7
Fellowship training		
Yes	28	68
No	13	32
Location of fellowship training		
Canada	17	61
United States	7	25
Britain or Ireland	2	7
France	2	7
Years treating lung cancer		
≤5	12	29
6–10	15	37
10–20	11	27
20–30	3	7
Locally advanced or metastatic nsclc cases annually		
≤20	4	10
21–50	9	22
51–100	16	39
>100	12	29
Practice setting		
University-affiliated	32	78
Community-based	9	22
Geographic location		
Western Canada	9	22
Ontario	21	51
Quebec	6	15
Atlantic Canada	5	12

**TABLE III:** Treatment recommendation for the case scenarios presented in Table I (*N* = 41)

Scenario			Respondents	
			(*n*)	(%)
Scenario A	Asymptomatic stage iiib	Immediate rt	9	22
		Delayed rt	32	78
Scenario B	Bulky stage iiib	rt, then chemotherapy	4	10
		Chemotherapy, then rt	5	12
		Concurrent chemoradiation	22	54
		rt alone	8	20
		Chemotherapy alone	1	2
		Best supportive care	1	2
Scenario C	Symptomatic stage iv	ebrt	22	54
		Brachytherapy	1	2
		Brachytherapy, then ebrt	2	5
		Endobronchial therapy, then ebrt	4	10
		ebrt, then chemotherapy	10	24
		Best supportive care	2	5

rt = radiation therapy; ebrt = external-beam rt.

**TABLE IV:** Management and perceptions for the three case scenarios presented in Table I

Variable	Responses [*n/N* (%)]		
	Scenario A	Scenario B	Scenario C
Treatment intent			
Radical	na	25/41 (61)	na
Palliative	na	16/41 (39)	na
Aim			
Prolong survival	—	22/41 (54)	—
Prevent symptoms	8/9 (89)	3/41 (7)	—
Relieve symptoms	1/9 (11)	15/41 (37)	41/41 (100)
Other	—	1/41 (2)	—
Estimated wait time from decision to rt start (weeks)			
>1	4/8 (50)^a^	5/39 (13)	8/39 (21)
1–2	3/8 (38)	23/39 (59)	16/39 (41)
2–4	1/8 (13)	10/39 (26)	14/39 (36)
>4	—	1/39 (3)	1/39 (3)
Median survival (months)			
<6	11/41 (27)	12/41 (29)	29/41 (71)
6–12	25/41 (61)	24/41 (59)	11/41 (27)
12–18	4/41 (10)	4/41 (10)	1/41 (2)
>18	1/41 (2)	1/41 (2)	—
Impact on median survival (months)			
rt			
None	30/41 (73)	5/39 (13)^b^	28/41 (68)
<3	7/41 (17)	24/39 (62)	12/41 (29)
3–6	4/41 (10)	10/39 (26)	1/41 (2)
Delayed rt			
None	31/41 (78)	na	na
<3	8/41 (20)	na	na
3–6	1/41 (2)	na	na
Chemotherapy			
None	na	14/39 (36)^b^	14/41 (34)
<3	na	19/39 (49)	24/41 (59)
3–6	na	6/39 (15)	3/41 (7)
Chemoradiation			
None	na	3/41 (7)	20/41 (49)
<3	na	9/41 (22)	16/41 (39)
3–6	na	13/41 (32)	4/41 (10)
>6		16/41 (39)	1/41 (2)

a No answer from 1 respondent.

b No answer from 2 respondents.

rt = radiation therapy.

**TABLE V:** Radiotherapy dose and fractionation for the case presented as Scenario B in Table I^a^

Respondents (n)	Dose and fractionation (Gy / fractions / weeks)	Stated intent
2	20 / 5 / 1	Palliative
7	30 / 10 / 2	Palliative
1	36 / 13 / 2.5	Palliative
2	40 / 15 / 3	Palliative
1	50 / 20 / 4	Palliative
1	50 / 25 / 5	Radical
11	60 / 30 / 6	1 Palliative
		10 Radical
11	66 / 33 / 6.5	Radical
1	60–66 / 30–33 / 6–6.5	Radical
1	63 / 34 / 7	Radical

a Concurrent chemoradiation was chosen by 1 respondent, but the dose was not specified.
